# Tomato Fruit Detection and Counting in Greenhouses Using Deep Learning

**DOI:** 10.3389/fpls.2020.571299

**Published:** 2020-11-19

**Authors:** Manya Afonso, Hubert Fonteijn, Felipe Schadeck Fiorentin, Dick Lensink, Marcel Mooij, Nanne Faber, Gerrit Polder, Ron Wehrens

**Affiliations:** ^1^Wageningen University and Research, Wageningen, Netherlands; ^2^Enza Zaden, Enkhuizen, Netherlands

**Keywords:** deep learning, phenotyping, agriculture, tomato, greenhouse

## Abstract

Accurately detecting and counting fruits during plant growth using imaging and computer vision is of importance not only from the point of view of reducing labor intensive manual measurements of phenotypic information, but also because it is a critical step toward automating processes such as harvesting. Deep learning based methods have emerged as the state-of-the-art techniques in many problems in image segmentation and classification, and have a lot of promise in challenging domains such as agriculture, where they can deal with the large variability in data better than classical computer vision methods. This paper reports results on the detection of tomatoes in images taken in a greenhouse, using the MaskRCNN algorithm, which detects objects and also the pixels corresponding to each object. Our experimental results on the detection of tomatoes from images taken in greenhouses using a RealSense camera are comparable to or better than the metrics reported by earlier work, even though those were obtained in laboratory conditions or using higher resolution images. Our results also show that MaskRCNN can implicitly learn object depth, which is necessary for background elimination.

## 1. Introduction

Tomatoes are an economically important horticultural crop and the subject of research in seed development to improve yield. As with many other crops, harvesting is a labor intensive task, and so is the manual measurement of phenotypic information. In recent years there has been great and increasing interest in automating agricultural processes like harvesting (Bac et al., 2014), pruning (Paulin et al., 2015), or localized spraying (Oberti et al., 2016). This has stimulated the development of image analysis and computer vision methods for the detection of fruits and vegetables. Since imaging is a quick and non-destructive way of measurement, detection of fruits, both ripe and unripe, and other plant traits using computer vision is also useful for phenotyping (Minervini et al., 2015; Das Choudhury et al., 2019) and yield prediction. The number of fruits during plant growth is an important trait not only because it is an indicator of the expected yield, but is also necessary for certain crops such as apple, where yield must be controlled to avoid biennial tree stress.

Compared to laboratory settings, greenhouses can be challenging environments for image analysis, as they are often optimized to maximize crop production thereby imposing restrictions on the possible placement of a camera and thereby its field of view. Further, variation in the colors or brightness of the fruits can be encountered over different plants of the same crop, over time for the same plant, over images of the same plant from different camera positions, etc (Bac, 2015; Barth, 2018). Repeated measurements are difficult because of ongoing work, changing circumstances such as lighting conditions, and a generally unfriendly atmosphere for electronic equipment.

Most methods for detecting and counting fruits, including tomatoes, have used colorspace transformations in which the objects of interest stand out, and extraction of features such as shape and texture (Gomes and Leta, 2012; Gongal et al., 2015). In most of these works, the discriminative features were defined by the developers, rather than learnt by algorithms. Computer vision solutions based on hand crafted features may not be able to cope with the level of variability commonly present in greenhouses (Kapach et al., 2012; Zhao et al., 2016a). Deep Convolutional Neural Networks (CNNs) are being used increasingly for image segmentation and classification due to their ability to learn robust discriminative features and deal with large variation (LeCun et al., 2015). They however require large annotated datasets for training.

The various flavors of deep learning in computer vision include (i) image classification, in which an image is assigned one label (Krizhevsky et al., 2012), (ii) semantic segmentation, in which each pixel is assigned a label (Long et al., 2015; Chen et al., 2018), and (iii) object detection, which assigns a label to a detected object, thereby providing the location of individual objects (Girshick, 2015; Ren et al., 2015; Girshick et al., 2016). Deep learning is being increasingly used in the domain of agriculture and plant phenotyping (Kamilaris and Prenafeta-Boldú, 2018; Jiang and Li, 2020). Classification at the level of the entire image has been used for detecting diseases (Mohanty et al., 2016; Ramcharan et al., 2019; Toda and Okura, 2019; Xie et al., 2020) or for identifying flowers (Nilsback and Zisserman, 2006). Semantic segmentation and object detection are more relevant for our problem of detecting fruits, and their use in agriculture will be briefly reviewed in the following sub-section.

### 1.1. Related Work

Object detection is the most informative instance of deep learning for the detection of fruits, but also requires more complex training data. Region based convolutional neural networks (R-CNNs) combine the selective search method (Uijlings et al., 2013) to detect region proposals (Girshick et al., 2016), and were the basis for the Fast-RCNN (Girshick, 2015), and Faster-RCNN (Ren et al., 2015) methods. The *You Only Look Once* (YOLO) (Redmon et al., 2016) detector applies a single neural network to the full image, dividing the image into regions and predicts bounding boxes and probabilities for each region, and is faster than Fast-RCNN which applies the model to an image at multiple locations and scales. These methods provide bounding-box segmentations of objects of interest, which does not directly convey which pixels belong to which object instance, especially when there are overlapping or occluding objects of the same class.

Mask-RCNN (He et al., 2017) provides a segmentation of both, the bounding box and pixel mask for each object. It uses a CNN architecture such as ResNet (He et al., 2016) as the backbone, which extracts feature maps, over which a region proposal network (RPN) sliding window is applied to calculate region proposals, which are then pooled with the feature maps. Finally, a classifier is applied over each pooled feature map resulting in a bounding box prediction corresponding to an instance of the particular class. The scheme until this point is the same as FasterRCNN. Mask-RCNN applies an additional CNN on the aligned region and feature map to obtain a mask for each bounding box.

In the domain of agriculture, earlier work on detecting fruits of various crops used “classical" machine vision techniques, involving detection and classification based on hand-crafted features (Song et al., 2014). In Brewer et al. (2006), post-harvest images of tomatoes were analyzed for phenotypic variation in fruit shape. The individual tomatoes were placed on a dark background which made the segmentation simple, and the fruit perimeters were then extracted.

A Support Vector Machine (SVM) binary classifier applied on different regions of an image, followed by fusing the decisions was proposed for detecting tomatoes in Schillaci et al. (2012), but this method suffers from too many false positives (low precision). In Zhao et al. (2016b), a pixel level segmentation method for ripe tomatoes was presented, based on fusing information from the Lab and YIQ colorspaces which emphasize the ripe tomatoes, followed by an adaptive threshold. In Zhang and Xu (2018), a pixel level fruit segmentation method was proposed, which assigns initial labels using conditional random fields and Latent Dirichlet Allocation, and then relates the labels for the image at different resolutions. Haar features from a colorspace transform followed by adaboost classification was used in Zhao et al. (2016c) for detecting ripe tomatoes. However, this method tried to fit circles, and did not try to accurately detect individual object masks.

A method for counting individual tomato fruits from images of a plant growing in a lab setting was presented in Yamamoto et al. (2014). This method used decision trees on color features to obtain a pixel wise segmentation, and further blob-level processing on the pixels corresponding to fruits to obtain and count individual fruit centroids. This method reported an overall detection precision of 0.88 and recall of 0.80.

In Hannan et al. (2009), oranges were counted from images in an orchard using adaptive thresholding on colorspace transforms, followed by sliding windows applied on the segmented blob perimeters to fit circles, with voting based on the fitted centroids and radii. A method for ripe sweet pepper detection and harvesting was proposed in Bac et al. (2017), that used detection of red blobs from the normalized difference of the red and green components, followed by a threshold number of pixels per fruit.

More recent works use deep learning, in its various flavors (Tang et al., 2020). A 10 layer convolutional neural network was used in Muresan and Oltean (2018) for classifying images of individual fruits including tomatoes, in a post-harvest setting. In Barth et al. (2017, 2018) a pixel wise segmentation of sweet pepper fruits and other plant parts was presented based on training the VGG network (Simonyan and Zisserman, 2014) on synthetic but realistic data. Deepfruits (Sa et al., 2016) is a detection method for fruits such as apples, mangoes, and sweet peppers, which adapts FasterRCNN to fuse information from RGB and Near-Infrared (NIR) images.

Another work on tomato plant part detection (Zhou et al., 2017) used a convolutional neural network with an architecture similar to VGG16 (Simonyan and Zisserman, 2014) and a region proposal network method similar to Fast-RCNN (Girshick, 2015) and obtained a mean average precision of 0.82 for fruits. It must be noted that (Zhou et al., 2017) is written in Mandarin, with an abstract in English. FasterRCNN object detection was also used in Fuentes et al. (2018) to detect diseased and damaged parts of a tomato plant. A single shot detection architecture based on a grid search was proposed in Bresilla et al. (2019) to detect bounding boxes of apples and pears from images of their trees. In Rahnemoonfar and Sheppard (2017), a modified version of the Inception-ResNet architecture was proposed and trained with a synthetic dataset of red circular blobs, and was able to detect tomato fruits with a prediction accuracy of 91 % on a dataset of images obtained from Google images mainly of cherry tomato plants.

Finally, MaskRCNN has been applied for the segmentation of *Brassica oleracea* (e.g., Broccoli, Caulifower) (Jiang et al., 2018) and leaves (Ward et al., 2018). In Santos et al. (2020), a method was presented for detecting and tracking grape clusters in images taken in vineyards, based on MaskRCNN for the detection of individual grape bunches and structure from motion for 3D alignment of images thereby enabling their mapping across images.

For quick reference, the above referenced methods are summarized in Table 1.

**Table 1:** Summary of metrics reported in related work on fruit detection.

**Work**	**Crop/plant**	**Method**	**Reported metrics (maximum/best value is 1.0)**
Hannan et al. (2009)	Orange	Adaptive thresholding + circle fitting	recall: 0.90, false detection rate: 0.04
Schillaci et al. (2012)	Tomato	Support Vector Machine based classifier	Precision: 0.44
Yamamoto et al. (2014)	Tomato	Decision tree + color, shape, texture classifier	recall: 0.80, precision: 0.88
Zhao et al. (2016b)	Tomato	Colorspace fusion + adaptive threshold	Recall (ripe only): 0.93
Sa et al. (2016)	Sweet pepper	Variant of FasterRCNN	F1: 0.84
Zhou et al. (2017)	Tomato	Variant of FasterRCNN	Avg Precision for fruits: 0.82, flowers: 0.85, stems: 0.54
Rahnemoonfar and Sheppard (2017)	Tomato	CNN trained on synthetic data	prediction accuracy: 0.91
Zhang and Xu (2018)	Tomato	Multi resolution Conditional Random Field	pixel level segmentation accuracy: 0.99
Barth (2018)	Sweet pepper	Semantic segmentation using VGG	pixel level IOU: 0.52
Bresilla et al. (2019)	Apple, pear	Grid search based single shot detection	F1 for apple: 0.9, pear: 0.87
Santos et al. (2020)	Grape	MaskRCNN + structure from motion	bunch level F1: 0.91

### 1.2. Contributions

In this work, we apply MaskRCNN for the detection of tomato fruits from images taken in a production greenhouse, using Intel RealSense cameras. We report results on the detection of tomatoes from these images, using MaskRCNN trained with images in which foreground fruits are annotated. After inference, we apply a post-processing step on the segmentation results, to further get rid of background fruit that may have been detected as foreground.

In summary, in this paper, we try to answer the following questions

1. Can we detect tomato fruits in real life practical settings?
2. Can state-of-the-art results be achieved for detecting tomato fruits using MaskRCNN?

It must be noted that we deal with images taken in a greenhouse, which are more difficult than laboratory (Yamamoto et al., 2014) or post-harvest (Muresan and Oltean, 2018) settings and we use RealSense cameras which are less expensive than the point and shoot camera used in Yamamoto et al. (2014), and have a lower resolution than the High Definition one used in Zhou et al. (2017).

## 2. Materials and Methods

### 2.1. Dataset

**Figure 1:**
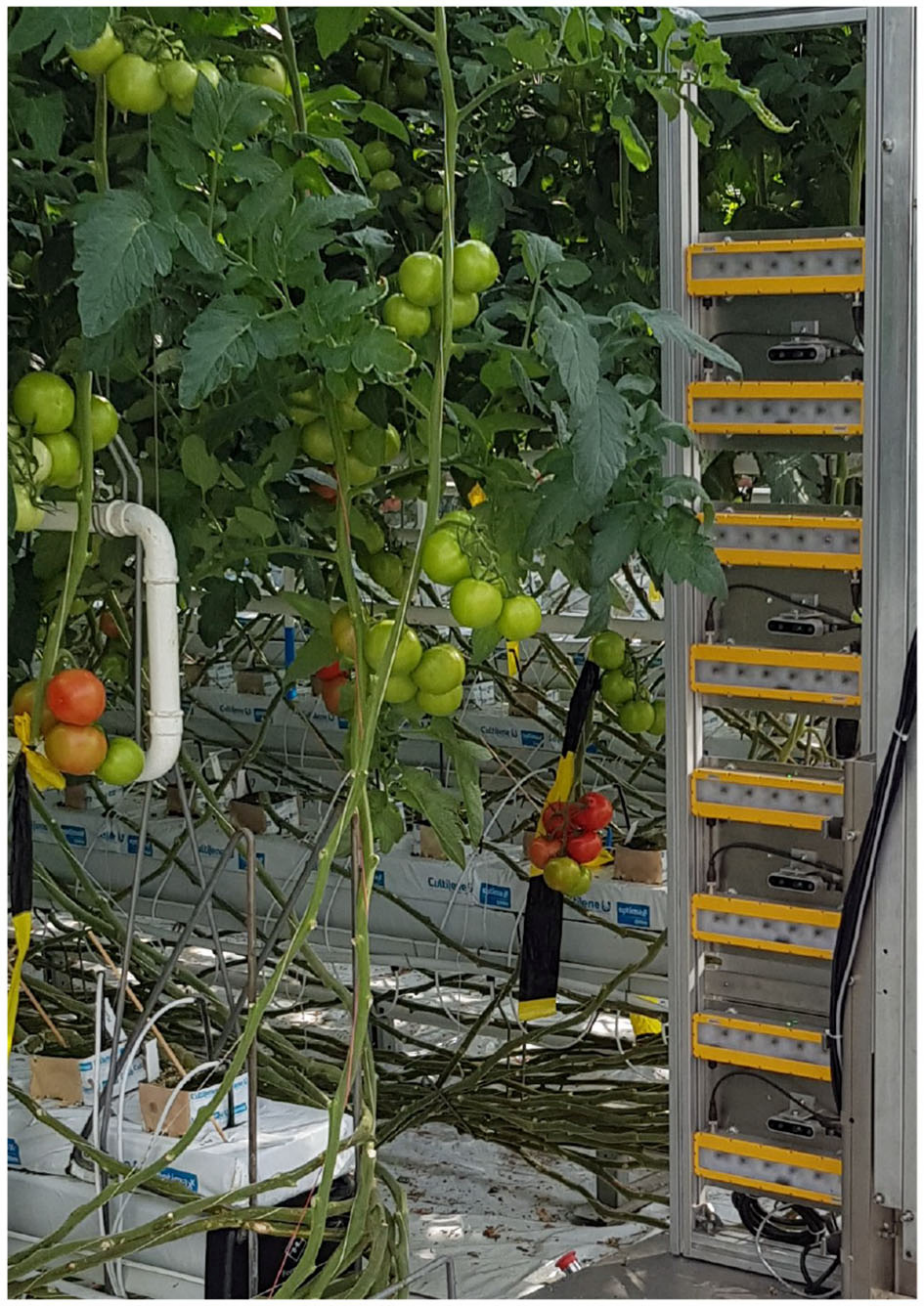
Imaging system consisting of four RealSense D435 cameras, mounted on the autonomous robot.

The robot and vision system were tested in Enza Zaden's production greenhouse in Enkhuizen, The Netherlands. The images were acquired with 4 Intel Realsense D435 cameras, mounted on a trolley that moves along the heating pipes of the greenhouse. The cameras are placed at heights of 930, 1,630, 2,300, and 3,000 mm from the ground, and are in landscape mode. They are roughly at a distance of 0.5 m from the plants. This setup is shown in Figure 1. With 4 cameras, an entire plant can be covered in one image acquisition event. Previously a robot equipped with a moving camera was used, but that took much more time for image acquisition and was much less practical, and the relative positions of the cameras was unstable.

The RealSense cameras were configured to produce pixel aligned RGB and depth images, of size 720 × 1280. The images were acquired at night, to minimize variability in lighting conditions due to direct sunlight or cloud cover, on 3 different dates at the end of May, and the first half of June 2019. In this work, we focus on fruit detection at the level of each individual image, and therefore, registration of the images at different camera heights and over nearby positions is not addressed in this paper.

A total of 123 images were manually annotated by a group of volunteers using the labelme annotation tool^1^. This tool allows polygons to be drawn around visible fruit, or even a circle in case the fruit is almost spherical. Due to occlusions, the same fruit may have multiple polygons, corresponding to disjoint segments. Thus, we obtain not just the bounding box, but also the pixels corresponding to each fruit. Only the tomatoes which belong to the plant in the row being imaged, i.e., the foreground, are annotated. The annotations were visually inspected and corrected fir missing foreground fruit, or incorrectly annotated background fruit. A Matlab script was used to merge the annotations saved from labelme into a JSON file according to the Microsoft COCO format^2^.

This set of images was randomly split into a training set (two thirds, 82 images) and a test set (one third, 41 images). It was ensured that annotations for images at different camera heights were included in both the training and test sets. Since the annotators only labeled fruits without specifying ripeness, to be able to work with two classes–ripe/red fruits and unripe/green fruits, we apply a post-processing step in Matlab to generate ground truth annotations with two classes ripe and unripe. The chromaticity map of the color image was calculated, and the chromaticity channel values over the respective fruit's pixels are compared. If the red channel exceeds 1.4 times the green channel of the chromaticity mapping for a majority of the fruit's pixels, it was assigned the label ripe, and if not, unripe. This cutoff factor was determined empirically.

**Figure 2:**
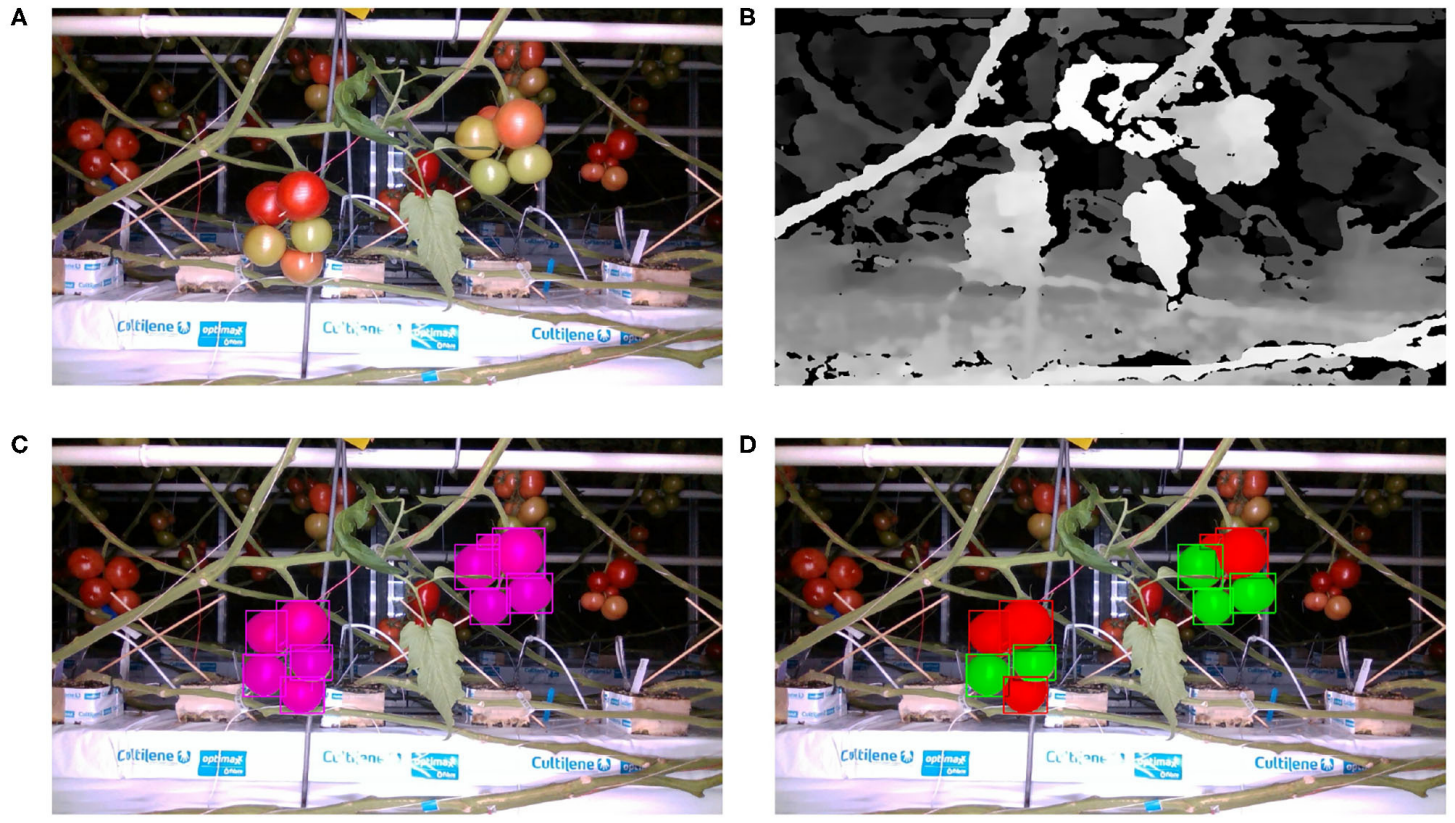
RealSense camera images example: **(A)** RGB image, **(B)** depth image, **(C)** RGB image overlaid with one class ground truth, **(D)** RGB image overlaid with 2 class ground truth.

A breakdown of the numbers of images and fruits by camera position and training/test set is presented in Table 2. Figure 2 shows an example of an RGB image from the RealSense camera, its corresponding depth, and the RGB image with the ground truth for the single fruit class and two ripeness classes overlaid.

**Table 2:** Breakdown of ground truth annotations by camera height.

**Camera**	**Training set**				**Test set**			
**Height**	**Images**	**Fruits**	**Red**	**Green**	**Images**	**Fruits**	**Red**	**Green**
Overall	83	1,074	365	709	40	538	176	362
930 mm	20	73	58	15	9	14	9	5
1,630 mm	27	308	177	131	13	166	101	65
2,300 mm	28	555	129	426	14	284	62	222
3,000 mm	8	138	1	137	4	74	4	70

### 2.2. Software and Setup

The MaskRCNN algorithm (He et al., 2017) has different implementations available, the best known being Detectron, Facebook AI Research's implementation (Girshick et al., 2018) and the Matterport implementation. Detectron is built on the Caffe2/PyTorch deep learning framework, whereas the Matterport version is built on Tensorflow. We opted for Detectron because it uses a format for annotated data, which is easier for dealing with occlusions and disjoint sections of an object.

It was installed on a workstation with an NVIDIA GeForce GTX 1080 Ti 11GB GPU, 12 core Intel Xenon E5-1650 processor and 64GB DDR4 RAM, running Linux Mint 18.3, supported by CUDA 9.0.

### 2.3. Architectures

The ResNet architecture (He et al., 2016) (50 and 101 layer versions) and ResNext (Xie et al., 2017) (101 layers, cardinality 64, bottleneck width 4) were used in our experiments. These architectures were pre-trained on the ImageNet-1K dataset (Russakovsky et al., 2015), with pre-trained models available from the Detectron model zoo.

### 2.4. Training Settings

MaskRCNN uses a loss function which is of the sum of the classification, bounding box, and mask losses. The Stochastic Gradient Descent (SGD) optimizer is used by default, and this was not changed. ℓ_2_ regularization was used on the weights, with a weight decay factor of 0.001. A batch size of 1 image was used throughout to avoid memory issues since only 1 GPU was used. No data augmentation was used. The optimal values of the learning rates were empirically determined to be 0.0025 for ResNet50, 0.001 for ResNet101, and 0.01 for ResNext101. The training was run for 2,00,000 iterations.

### 2.5. Post-processing of Segmentation Results

The segmentations produced by MaskRCNN were post-processed to discard fruits from the background that may have been picked up as foreground. A Matlab script reads an image, its corresponding depth image, and its segmented objects. For each segmented object, the median depth value over the pixels corresponding to its mask is computed. Since the depth encoding in the RealSense acquisition software uses higher depth values for objects closer to the camera, a detected fruit is considered to be in the foreground if its median depth exceeds a certain threshold. Since the perspectives of the cameras are different and the fact that the fruit trusses are often closer to the middle two cameras, the depth threshold up to which a pixel is considered to be in the foreground differs by camera. The empirically selected threshold values as a function of camera height are summarized in Table 3. The depth value is encoded in 8 bits, and therefore ranges from 0 to 255.

**Table 3:** Depth intensity foreground limits. Pixels with depth values above these cutoffs are considered foreground.

**Camera**	**Cut-off depth intensity**
930 mm	110
1,630 mm	120
2,300 mm	120
3,000 mm	50

### 2.6. Performance Evaluation

We evaluate the results of MaskRCNN on our validation set. A detected instance is considered a true positive if it has a Jaccard Index similarity coefficient also known as intersection-over-union (IOU) (He and Garcia, 2009; Csurka et al., 2013) of 0.5 or more with a ground truth instance. We also vary this threshold overlap with values of 0.25 (low overlap) and 0.75 (high overlap). The IOU is defined as the ratio of the number of pixels in the intersection to the number of pixels in the union. Those ground truth instances which did not overlap with any detected instance are considered false negatives. From these measures, the precision, recall, and F1 score were calculated,

(1) $$\begin{array}{l} \text{Precision}=\frac{\text{TP}}{\text{TP}+\text{FP}} \end{array}$$

(2) $$\begin{array}{l} \text{Recall}=\frac{\text{TP}}{\text{TP}+\text{FN}} \end{array}$$

(3) $$\begin{array}{l} \text{F1}=\frac{2\text{Precision }\times \text{ Recall}}{\text{Precision }+\text{ Recall}} \end{array}$$

where *TP* = the number of true positives, *FP* = the number of false positives, and *FN* = the number of false negatives.

For comparison, we also provide the results of our earlier work on detecting and counting tomatoes on the same dataset, which uses colorspace transforms and watershed segmentation, and detects roughly circular regions based on how closely the perimeters of detected regions can fit circles. This method was implemented in MVTec Halcon.

## 3. Results

### 3.1. Detection of Tomato Fruits in General

**Figure 3:**
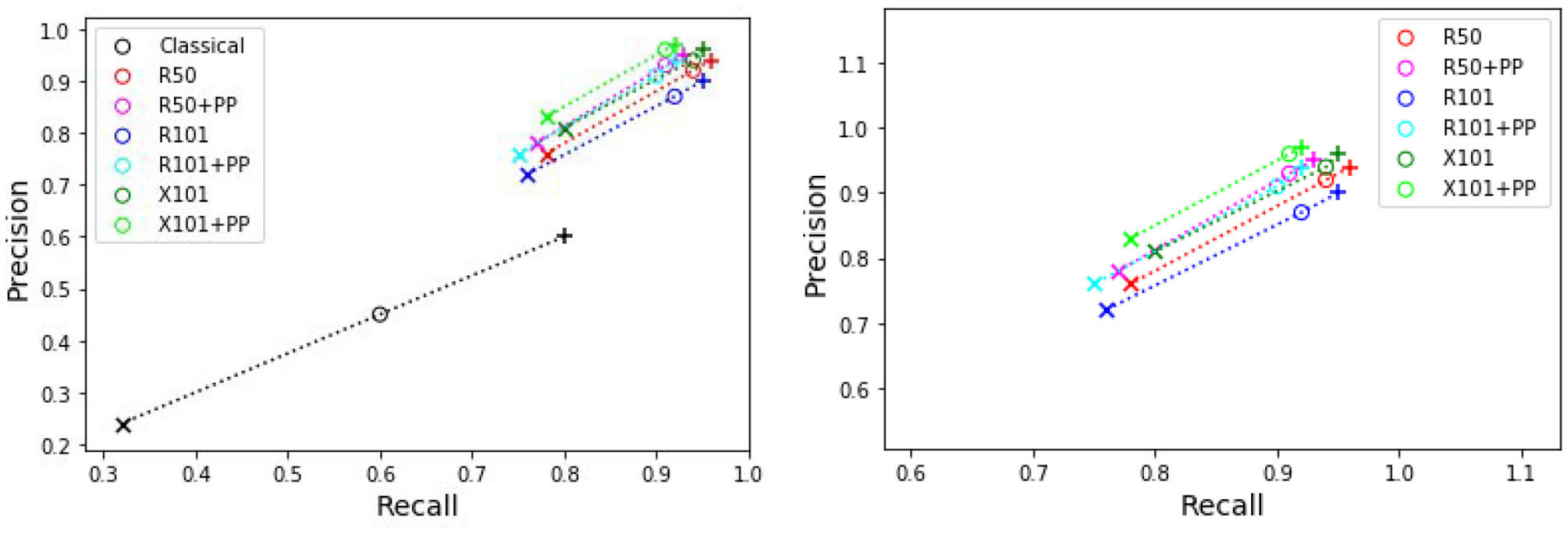
Plot of detection results on the test set using a model trained for a single fruit class. PP indicates depth post-processing. Each color corresponds to one method/architecture. Symbols +, o, and × represent overlap IoU thresholds of 25, 50, and 75 %, respectively. The results for each method with these IoU thresholds are linked by dashed lines. The zoomed in version of the scatter plot excluding the classical segmentation is shown in the right side.

**Figure 4:**
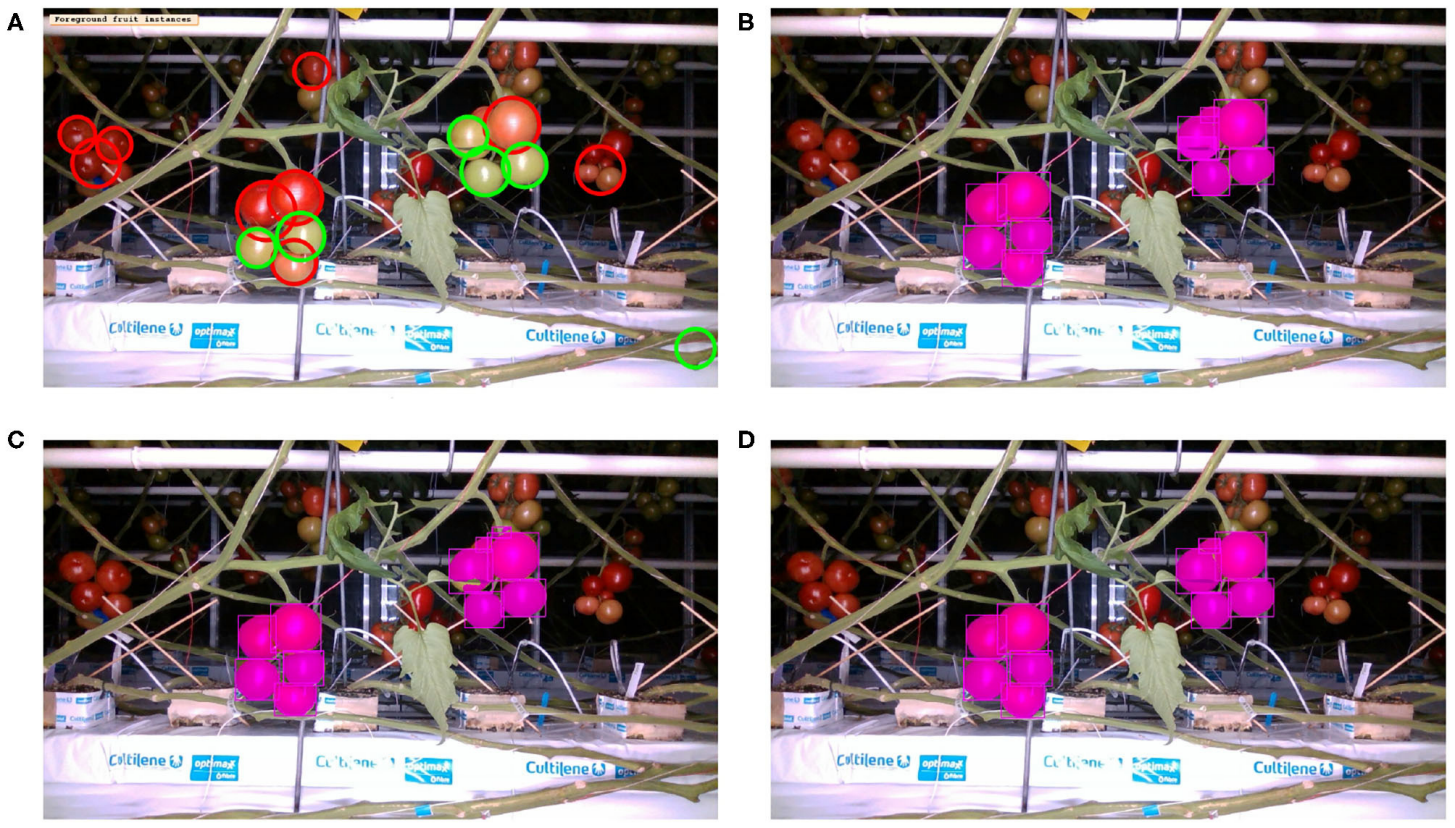
Inference with single fruit class: Image from Figure 2 overlaid with fruit detection using **(A)** Classical segmentation using colorspaces and shape, **(B)** MaskRCNN with R50 architecture, **(C)** MaskRCNN with R101, **(D)** MaskRCNN with X101.

Table 4 presents the precision and recall metrics on the test set for single class fruit detection, with different architectures and with a breakdown of these metrics over images from each of the 4 cameras. For visual comparison, we present these metrics as a 2D scatter plot, with the x-axis corresponding to the recall, and the y-axis to the precision, in Figure 3. Each color corresponds to inference results with one method/architecture. For each color, the symbols +, o, and × represent overlap IoU thresholds of 25, 50, and 75 %, respectively. Since ideally we would like both metrics to be close to 1, the best method is the one which is as much as possible to the top right corner. Figure 4 shows the results of detection using the classical segmentation method and using MaskRCNN, on the image from Figure 2.

**Table 4:** Summary of detection results on the test set using a model trained for a single fruit class.

**Network/**	**Camera**	**Overlap = 0.25**				**Overlap = 0.5**				**Overlap = 0.75**			
**algorithm**		**Precision**	**Recall**	**IoU**	**F1**	**Precision**	**Recall**	**IoU**	**F1**	**Precision**	**Recall**	**IoU**	**F1**
Classical	Overall	0.6	0.8	0.48	0.69	0.45	0.60	0.48	0.58	0.24	0.32	0.48	0.27
Segmentation													
	Overall	0.93	0.96	0.71	0.95	0.92	0.94	0.71	0.93	0.76	0.78	0.71	0.77
	1	0.87	0.93	0.82	0.9	0.87	0.93	0.82	0.9	0.8	0.86	0.82	0.83
R50	2	0.92	0.96	0.54	0.94	0.9	0.95	0.54	0.92	0.74	0.78	0.54	0.76
	3	0.97	0.99	0.87	0.98	0.95	0.98	0.87	0.96	0.82	0.84	0.87	0.83
	4	0.86	0.84	0.65	0.85	0.82	0.8	0.65	0.81	0.56	0.54	0.65	0.55
	Overall	0.95	0.93	0.7	0.94	0.93	0.91	0.7	0.92	0.78	0.77	0.7	0.77
	1	1	0.93	0.88	0.96	1	0.93	0.88	0.96	0.92	0.86	0.88	0.89
R50	2	0.94	0.95	0.54	0.95	0.92	0.93	0.54	0.93	0.76	0.77	0.54	0.77
+PP	3	0.98	0.99	0.87	0.98	0.97	0.98	0.87	0.97	0.83	0.84	0.87	0.84
	4	0.83	0.65	0.54	0.73	0.78	0.61	0.54	0.68	0.57	0.45	0.54	0.5
	Overall	0.9	0.95	0.7	0.92	0.87	0.92	0.69	0.9	0.72	0.76	0.69	0.74
	1	0.78	1	0.79	0.88	0.78	1	0.79	0.88	0.67	0.86	0.79	0.75
R101	2	0.88	0.97	0.53	0.92	0.83	0.91	0.53	0.87	0.7	0.78	0.53	0.74
	3	0.93	0.97	0.85	0.95	0.92	0.96	0.85	0.94	0.76	0.8	0.85	0.78
	4	0.86	0.81	0.64	0.83	0.81	0.77	0.64	0.79	0.6	0.57	0.63	0.58
	Overall	0.94	0.92	0.69	0.93	0.91	0.9	0.69	0.9	0.76	0.75	0.69	0.75
	1	1	1	0.89	1	1	1	0.89	1	0.86	0.86	0.89	0.86
R101	2	0.95	0.95	0.54	0.95	0.89	0.9	0.54	0.89	0.77	0.77	0.54	0.77
+PP	3	0.95	0.97	0.85	0.96	0.94	0.96	0.85	0.95	0.78	0.8	0.85	0.79
	4	0.83	0.65	0.54	0.73	0.79	0.62	0.54	0.7	0.62	0.49	0.54	0.55
	Overall	0.96	0.95	0.72	0.95	0.94	0.94	0.72	0.94	0.81	0.8	0.72	0.8
	1	0.93	1	0.87	0.97	0.93	1	0.87	0.97	0.87	0.93	0.87	0.9
X101	2	0.92	0.94	0.54	0.93	0.91	0.93	0.54	0.92	0.8	0.81	0.54	0.81
	3	0.98	0.98	0.88	0.98	0.98	0.97	0.88	0.97	0.84	0.83	0.87	0.84
	4	0.93	0.85	0.68	0.89	0.9	0.82	0.68	0.86	0.68	0.62	0.68	0.65
	Overall	0.97	0.92	0.71	0.94	0.96	0.91	0.71	0.93	0.83	0.78	0.71	0.81
	1	1	1	0.89	1	1	1	0.89	1	0.93	0.93	0.89	0.93
X101	2	0.95	0.92	0.54	0.94	0.94	0.91	0.54	0.92	0.83	0.81	0.54	0.82
+PP	3	0.99	0.98	0.88	0.98	0.99	0.97	0.88	0.98	0.85	0.83	0.88	0.84
	4	0.93	0.68	0.57	0.78	0.89	0.65	0.57	0.75	0.7	0.51	0.57	0.59

*Each row corresponds to inference results with one method/architecture. When depth post-processing is used, it is indicated by +PP*.

From Table 4 and Figure 3, it can be seen that the results of MaskRCNN using all the architectures are substantially better than those obtained using the Halcon based classical computer vision method. For all methods, we can notice that both precision and recall metrics are lower when the overlap threshold is 75 %, and the highest when this threshold is 25 %. This means that for a stricter matching criterion (higher IoU threshold), fewer detected fruit are being matched with an instance from the ground truth, leading to both metrics being lower. The architectures whose results are the closest to the top right corner are ResNet50 (R50) and ResNext101 (X101), for overlap threshold of both 25 and 50%.

For more visual results of tomato fruit detection with examples of images from each of the four cameras, please refer to the Supplementary Material.

### 3.2. Detection of Ripe and Unripe Fruits Separately

**Figure 5:**
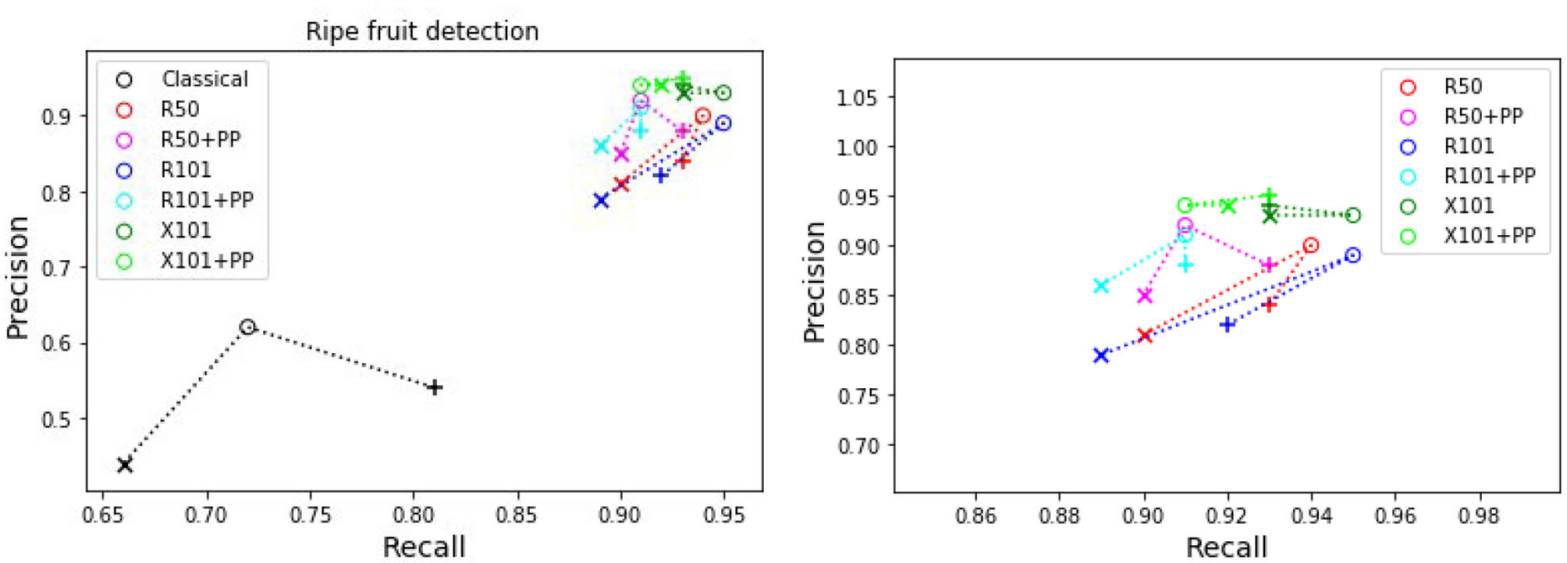
Plots of detection metrics for the two class case. Each color corresponds to one method/architecture. Symbols +, o, and × represent overlap IoU thresholds of 25, 50, and 75 %, respectively. The results for each method with these IoU thresholds are linked by dashed lines. The zoomed in version of the scatter plot excluding the classical segmentation is shown in the right side.

**Figure 6:**
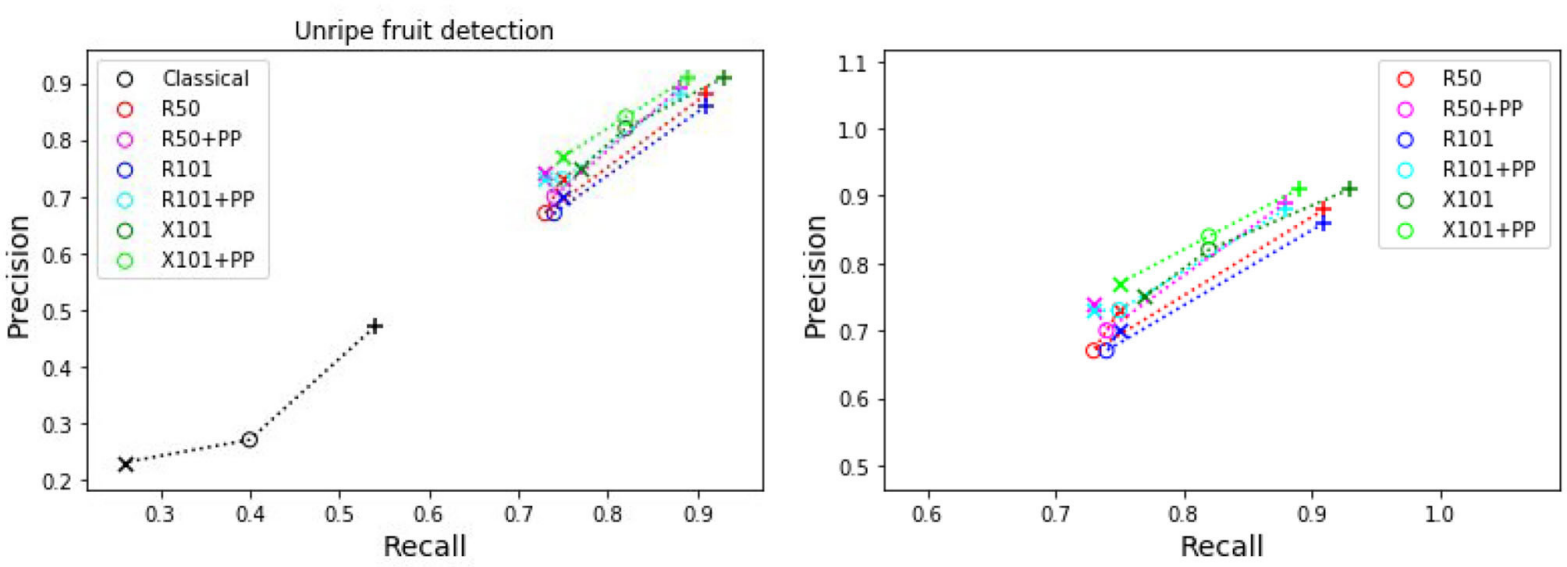
Plots of detection metrics for the two class case. Each color corresponds to one method/architecture. Symbols +, o, and × represent overlap IoU thresholds of 25, 50, and 75 %, respectively. The results for each method with these IoU thresholds are linked by dashed lines. The zoomed in version of the scatter plot excluding the classical segmentation is shown in the right side.

**Figure 7:**
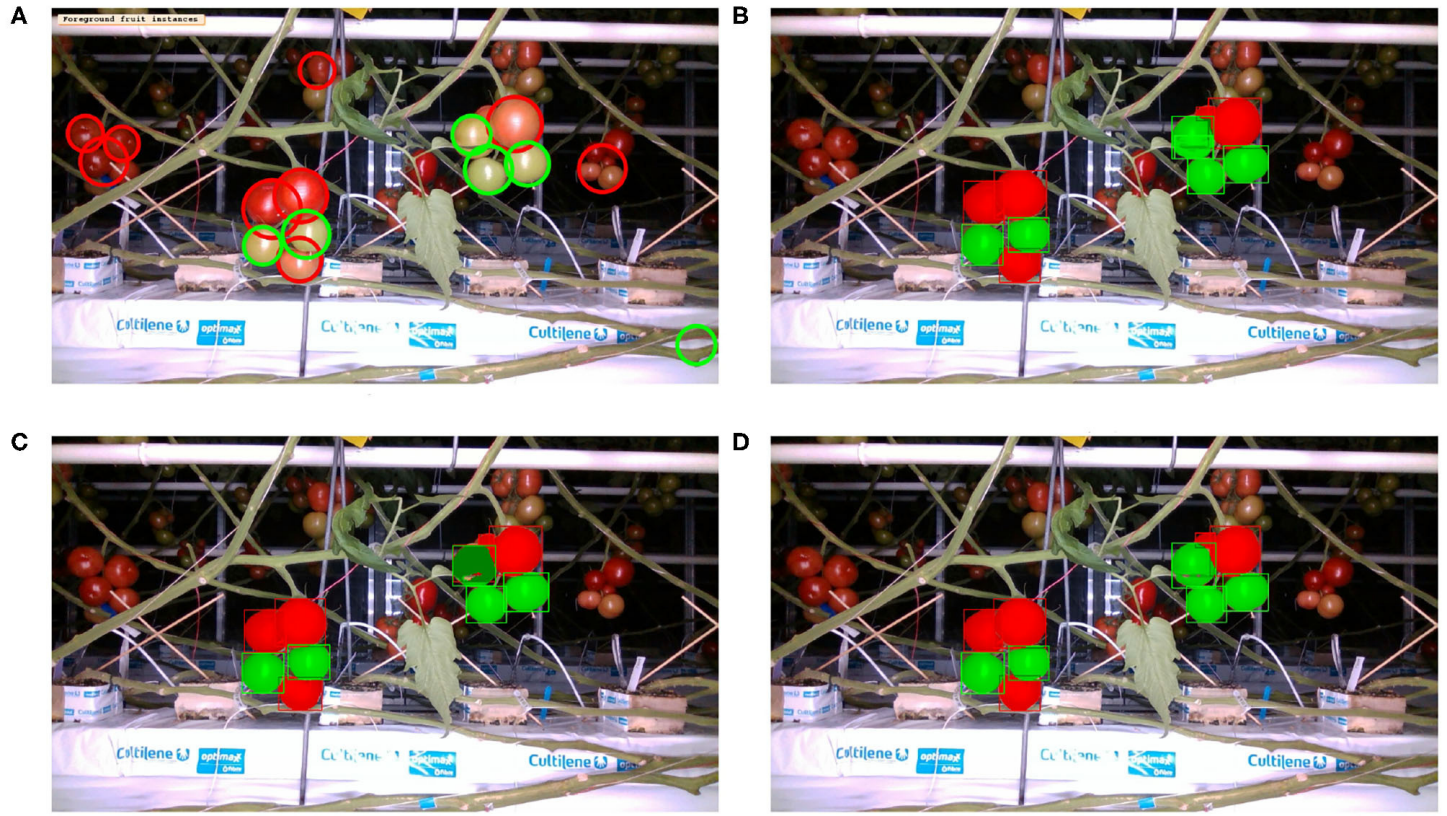
Inference with two ripeness classes: Image from Figure 2 overlaid with fruit detection using **(A)** classical segmentation based on colorspaces and circularity, **(B)** MaskRCNN with R50 architecture, **(C)** MaskRCNN with R101, **(D)** MaskRCNN with X101.

The precision and recall metrics for the two class case, obtained using the different architectures are presented in Table 5, along with the breakdown by camera, and shown as scatter plots in Figures 5, 6, for the red/ripe and green/unripe fruits, respectively. The color and symbol notations are the same as those in Figure 3. Figure 7 shows the detection results obtained for the same image from Figure 4.

**Table 5:** Summary of detection results on the test set using a model trained for a two fruit classes.

**Network/**	**Camera**	**overlap = 0.25**						**overlap = 0.5**						**overlap = 0.75**					
**algorithm**		**Red**			**Green**			**Red**			**Green**			**Red**			**Green**		
		**P**	**R**	**F1**	**P**	**R**	**F1**	**P**	**R**	**F1**	**P**	**R**	**F1**	**P**	**R**	**F1**	**P**	**R**	**F1**
classical	Overall	0.54	0.81	0.65	0.62	0.72	0.67	0.44	0.66	0.53	0.47	0.54	0.50	0.27	0.40	0.32	0.23	0.26	0.24
segmentation																			
	Overall	0.84	0.93	0.88	0.9	0.94	0.92	0.81	0.9	0.85	0.88	0.91	0.89	0.66	0.73	0.69	0.73	0.75	0.74
	1	0.7	0.78	0.74	1	1	1	0.7	0.78	0.74	1	1	1	0.7	0.78	0.74	0.8	0.8	0.8
R50	2	0.88	0.93	0.9	0.81	0.97	0.88	0.86	0.91	0.88	0.76	0.91	0.83	0.68	0.72	0.7	0.63	0.75	0.68
	3	0.82	0.94	0.88	0.95	0.98	0.96	0.77	0.89	0.83	0.93	0.96	0.94	0.66	0.76	0.71	0.8	0.83	0.81
	4	0.67	1	0.8	0.86	0.79	0.82	0.67	1	0.8	0.81	0.74	0.77	0.33	0.5	0.4	0.56	0.51	0.53
	Overall	0.88	0.93	0.9	0.92	0.91	0.91	0.85	0.9	0.87	0.89	0.88	0.88	0.7	0.74	0.72	0.74	0.73	0.73
	1	1	0.78	0.88	1	1	1	1	0.78	0.88	1	1	1	1	0.78	0.88	0.8	0.8	0.8
R50+PP	2	0.92	0.93	0.92	0.84	0.94	0.89	0.9	0.91	0.9	0.78	0.88	0.83	0.72	0.72	0.72	0.66	0.74	0.7
	3	0.82	0.94	0.88	0.96	0.98	0.97	0.77	0.89	0.83	0.95	0.96	0.95	0.66	0.76	0.71	0.82	0.83	0.82
	4	0.75	1	0.86	0.85	0.64	0.73	0.75	1	0.86	0.79	0.6	0.68	0.5	0.67	0.57	0.55	0.41	0.47
	Overall	0.82	0.91	0.86	0.89	0.95	0.92	0.79	0.89	0.84	0.86	0.91	0.88	0.66	0.74	0.7	0.7	0.75	0.72
	1	0.64	1	0.78	0.71	1	0.83	0.57	0.89	0.69	0.71	1	0.83	0.57	0.89	0.69	0.71	1	0.83
R101	2	0.84	0.9	0.87	0.79	0.97	0.87	0.83	0.89	0.86	0.74	0.91	0.82	0.67	0.71	0.69	0.6	0.74	0.66
	3	0.83	0.92	0.87	0.94	0.98	0.96	0.78	0.87	0.82	0.92	0.96	0.94	0.7	0.77	0.73	0.78	0.82	0.8
	4	0.67	1	0.8	0.85	0.81	0.83	0.67	1	0.8	0.79	0.76	0.77	0.5	0.75	0.6	0.52	0.5	0.51
	Overall	0.88	0.91	0.89	0.91	0.91	0.91	0.86	0.89	0.87	0.88	0.88	0.88	0.73	0.75	0.74	0.72	0.73	0.72
	1	1	1	1	1	1	1	0.89	0.89	0.89	1	1	1	0.89	0.89	0.89	1	1	1
R101+PP	2	0.91	0.89	0.9	0.81	0.92	0.86	0.91	0.89	0.9	0.77	0.88	0.82	0.73	0.71	0.72	0.64	0.72	0.68
	3	0.84	0.92	0.88	0.96	0.98	0.97	0.79	0.87	0.83	0.94	0.96	0.95	0.71	0.77	0.74	0.8	0.82	0.81
	4	0.75	1	0.86	0.82	0.66	0.73	0.75	1	0.86	0.77	0.61	0.68	0.75	1	0.86	0.52	0.41	0.46
	Overall	0.94	0.93	0.93	0.93	0.95	0.94	0.93	0.93	0.93	0.91	0.93	0.92	0.82	0.82	0.82	0.75	0.77	0.76
	1	1	0.89	0.94	0.83	1	0.91	1	0.89	0.94	0.83	1	0.91	1	0.89	0.94	0.83	1	0.91
X101	2	0.94	0.91	0.92	0.85	0.97	0.91	0.93	0.9	0.91	0.81	0.92	0.86	0.84	0.81	0.82	0.62	0.71	0.66
	3	0.92	0.97	0.94	0.96	0.98	0.97	0.92	0.97	0.94	0.94	0.96	0.95	0.8	0.84	0.82	0.82	0.84	0.83
	4	1	1	1	0.92	0.83	0.87	1	1	1	0.89	0.8	0.84	0.5	0.5	0.5	0.65	0.59	0.62
	Overall	0.95	0.93	0.94	0.94	0.91	0.92	0.94	0.92	0.93	0.91	0.89	0.9	0.84	0.82	0.83	0.77	0.75	0.76
	1	1	0.89	0.94	1	1	1	1	0.89	0.94	1	1	1	1	0.89	0.94	1	1	1
X101+PP	2	0.96	0.9	0.93	0.86	0.94	0.9	0.95	0.89	0.92	0.82	0.89	0.85	0.86	0.81	0.83	0.63	0.69	0.66
	3	0.92	0.97	0.94	0.97	0.98	0.97	0.92	0.97	0.94	0.95	0.96	0.95	0.8	0.84	0.82	0.83	0.84	0.83
	4	1	1	1	0.92	0.67	0.78	1	1	1	0.88	0.64	0.74	0.67	0.67	0.67	0.67	0.49	0.57

*Each row corresponds to inference results with one method/architecture. When depth post-processing is used, it is indicated by +PP*.

As before for the single class case, we can see in Figures 5, 6 that the MaskRCNN metrics are noticeably higher than those obtained using the classical segmentation, for both the ripe and unripe fruit classes. However, unlike the single class case, for the ripe fruits, the metrics obtained with an IoU threshold of 50 % are higher than those with 75 %, but the precision is lower when the threshold is 25 %. This means that for the ripe fruits, lowering the overlap threshold causes more detections to be considered false positives, which can be explained by ambiguity in defining the color cutoff to define the ground truth class. For the both ripe and unripe fruits, the metrics closest to the top right corner are those obtained using the ResNext101 (X101) architecture, while ResNet101 (R101) achieves a comparable recall for the ripe fruits.

## 4. Discussion

Comparing the visual results in Figures 4, 7 obtained using classical segmentation (hand-crafted features), the results of MaskRCNN have much fewer false positives and false negatives. It can also be seen in Figures 3, 5, 6 that MaskRCNN obtains considerably higher values of the precision and recall. Thus, MaskRCNN can better deal with the variability in the dataset than classical computer vision based on color and geometry.

Using MaskRCNN for detecting tomatoes on our data exceeds the metrics reported in previous work. The precision and recall values for MaskRCNN from Tables 4, 5, and Figures 3, 5, 6 exceed the values of precision 0.88 and recall 0.8 reported in Yamamoto et al. (2014), and the average precision of 0.82 reported in Zhou et al. (2017). Our recall values also consistently exceed the prediction accuracy of 0.91 reported in Rahnemoonfar and Sheppard (2017).

The ResNext 101 (X101) architecture is consistently better than the ResNet 50 and 101 layer architectures, as can be verified in Figures 3, 5, 6. It can be seen that the points corresponding to ResNext101 are the closest to the top right corner of the plot, and considering both precision and recall are always better than the other architectures.

With regards to the difference in detection performance over the four cameras, it can be seen from Tables 4, 5, that the metrics for the top most camera (number 4) are not as good as those of the other 3. This can be explained by there being more leaves toward the top of the plants, which make the detection of green fruits more difficult and also affect the depth thresholding. More illustrative examples can be found in the Supplementary Material, specifically Supplementary Figures 7–10 show images from the top most camera. Since there are very few ripe fruits at the height of camera 4, the metrics for the single class case are dominated by the unripe fruits. The ResNext 101 architecture again deals with this difference better than the other networks. In Table 4, without depth post-processing, the architecture obtains precision and recall values which are still comparable to or greater than those reported in Yamamoto et al. (2014) and Zhou et al. (2017). In practice the lowest camera is the most important one, since that is the height at which fruits are harvested. The top camera is used for forecasting and therefore does not necessarily need the same precision as the best camera. In addition, since the acquisition robot is automated, runs can be done every single night so that the chance of missing a fruit through obstruction is decreased quite significantly.

It can be seen from the results for both single and two class detection in Figures 3, 5, 6 that post-processing using depth to discard background false positives improves the precision but lowers the recall. This can be explained by the fact that the depth values from the RealSense depth image may have some errors, leading to some foreground fruit not being included when post-processing. Refer to Supplementary Figures 11–14 for some examples of these situations. But even without resorting to depth post-processing, the networks still learn the foreground well, since we are training with accurate foreground tomatoes through annotation by experts.

For ripeness, the metrics in Figure 5 are slightly worse for the red tomatoes. The ambiguity in defining the cut-off between the ripe and unripe classes along with the fact that our dataset contains almost twice as many unripe tomatoes as ripe ones, leads to more false positives for the ripe class than the unripe. It may therefore better to detect a single class fruits and then score a scale of ripeness.

On the basis of the results we report, we find that it is possible to robustly detect tomatoes from images taken in practical settings, without using complex and expensive imaging equipment. This can open the door to automating phenotypic data collection on a large scale, and can eventually be applied in automatic harvesting.

## 5. Conclusions and Future Work

In this work, deep learning instance detection using MaskRCNN was applied to the problem of detecting tomato fruits. Experimental results show that this approach works well for the detection of tomatoes in a challenging experimental setup and using a set of simple inexpensive cameras, which is of interest to practical applications such as harvesting and yield estimation.

In future work, we will address the integration of the results of fruit detection from individual images to the level of plots, to perform a comparison with harvested yield. Including the depth as an additional input layer to MaskRCNN may also be a possible way to try and improve the detection results. This would require some method of improving the depth image quality, such as Godard et al. (2017). Finally, the use of deep learning for the detection of other plant parts such as stems and peduncles, will also be addressed.

## Data Availability Statement

The dataset generated for this study is available on request to the corresponding author.

## Author Contributions

MA was involved in writing the bulk of the manuscript and in setting up the software and for running the deep learning experiments. HF was involved in preparing the setup for annotating the images, data analysis, and in reviewing the manuscript. FF was involved in optimizing the deep learning parameters that were used to obtain the results reported. DL, MM, and NF from Enza Zaden were responsible for running the robot that acquired images and provided the data and were involved in reviewing the manuscript. DL was also involved in the data analysis. GP's role was in developing the vision system and integrating it with the robot platform, in the data annotation, and provided a lot of input on the image analysis and on the manuscript. RW was the principal investigator of the project and was responsible for the overall project management, and the definition and conceptualization of the extraction of phenotypic information from the images. He also contributed a lot to the organization of the manuscript. All authors contributed to the article and approved the submitted version.

## Conflict of Interest

The authors declare that the research was conducted in the absence of any commercial or financial relationships that could be construed as a potential conflict of interest.

## Footnotes

^1^https://github.com/wkentaro/labelme

^2^http://cocodataset.org/#format-data
