# Supplementary material for: Tomato Fruit Detection and Counting in Greenhouses Using Deep Learning
Source: Front Plant Sci. 2020 Nov 19;11:571299. doi: 10.3389/fpls.2020.571299 (PMC7717966; doi:10.3389/fpls.2020.571299)
Supplement: Supplementary file 1 [file Data_Sheet_1.pdf]

Supplementary Material for paper Tomato Fruit Detection and Counting in Greenhouses using Deep Learning

1 SUPPLEMENTARY TABLES AND FIGURES

1.1 Examples of Detection

Some additional results of tomato detection are presented here. Figures S1,S3,S5, S7, and S9 show the results of detection using the classical segmentation method and using MaskRCNN for the single fruit class. The results of detection for the two ripeness classes on the same images are presented in Figures S2,S4,S6, S8, and S10.

The respective camera numbers are indicated in each example, 1 being the lowest and 4 being the top-most. Figures S7, S9, S8, and S10 show results of detection from the top-most camera, and it can be seen that more leaves are present.

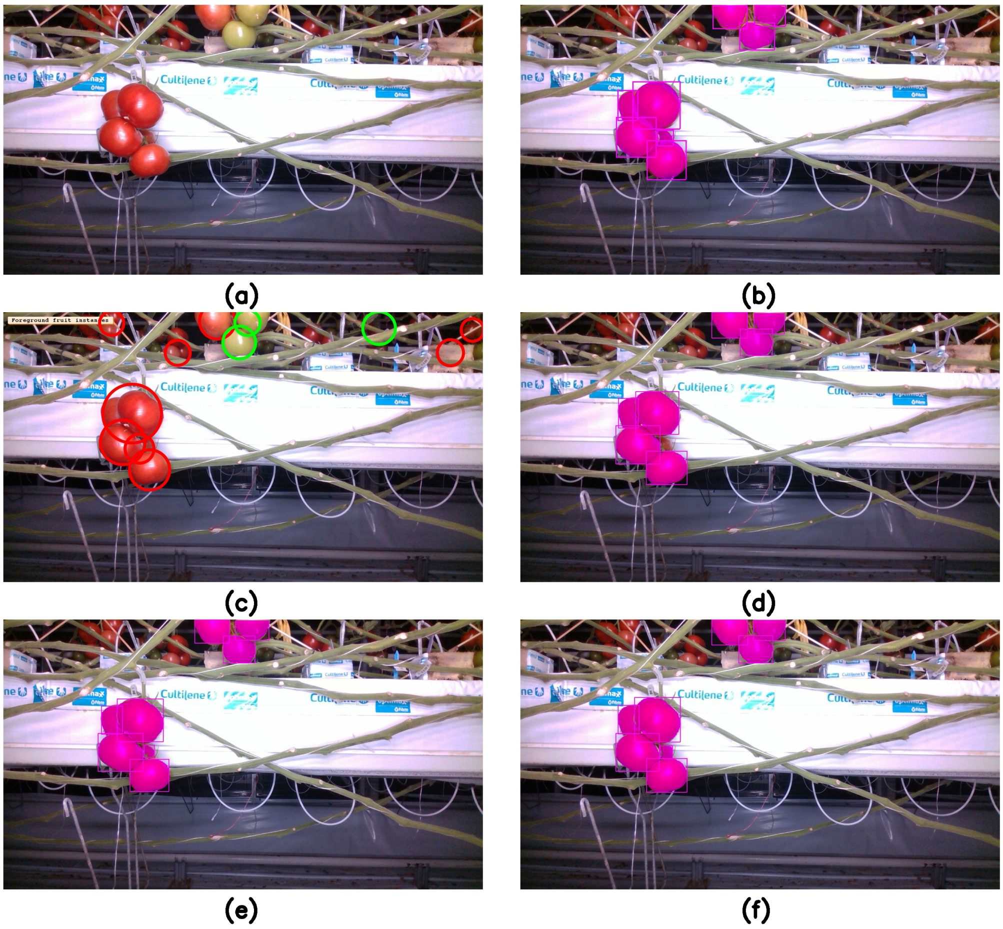

Figure S1. Camera 1 (height 930 mm), inference with single fruit class: (a) RGB image; (b) image with overlaid ground truth; (c-f) image overlaid with fruit detection using (c) Classical segmentation using colorspaces and shape, (d) MaskRCNN with R50 architecture, (e) MaskRCNN with R101, (f) MaskRCNN with X101.

1.2 Comparison with and without Post-processing

Figures S11, S12, S13, and S14 show the results of detection with and without post processing to discard background fruits.

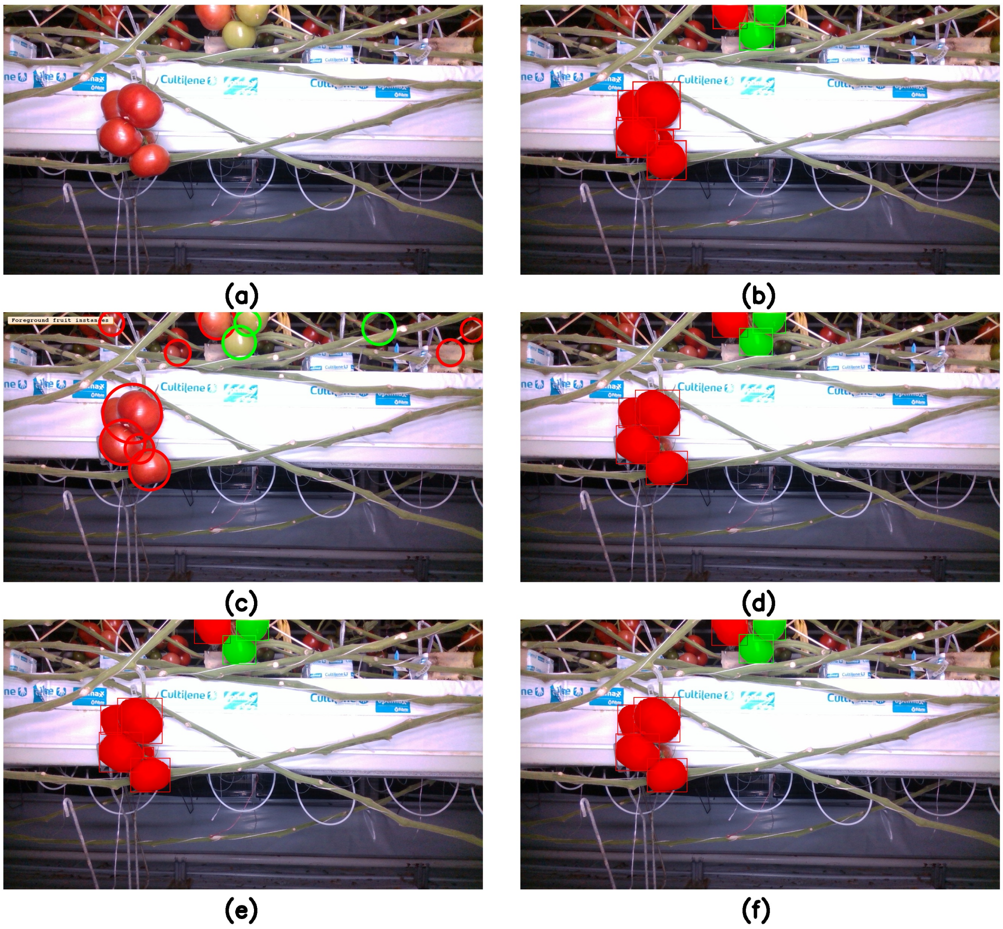

Figure S2. Camera 1 (height 930 mm), inference with two ripeness classes: (a) RGB image; (b) image with overlaid ground truth; (c-f) image overlaid with fruit detection using (c) Classical segmentation using colorspaces and shape, (d) MaskRCNN with R50 architecture, (e) MaskRCNN with R101, (f) MaskRCNN with X101.

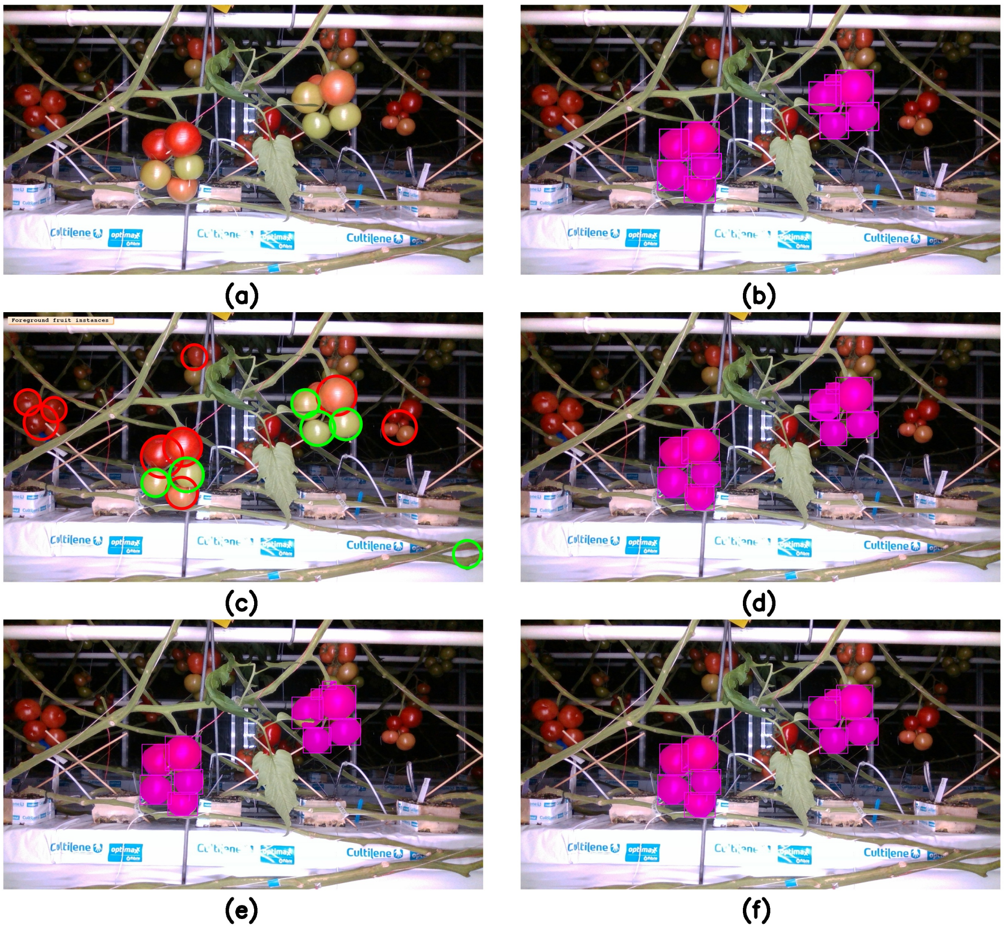

Figure S3. Camera 2 (height 1630 mm), inference with single fruit class: (a) RGB image; (b) image with overlaid ground truth; (c-f) image overlaid with fruit detection using (c) Classical segmentation using colorspaces and shape, (d) MaskRCNN with R50 architecture, (e) MaskRCNN with R101, (f) MaskRCNN with X101.

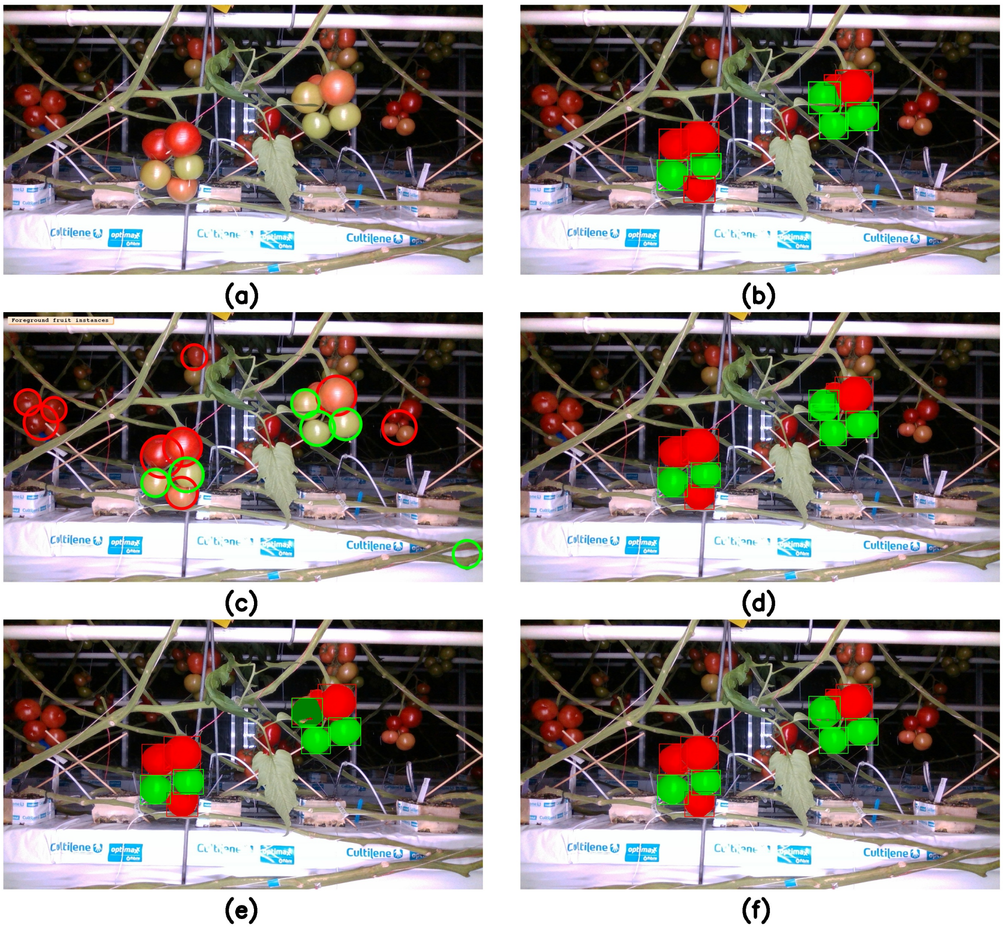

Figure S4. Camera 2 (height 1630 mm), inference with two ripeness classes: (a) RGB image; (b) image with overlaid ground truth; (c-f) image overlaid with fruit detection using (c) Classical segmentation using colorspaces and shape, (d) MaskRCNN with R50 architecture, (e) MaskRCNN with R101, (f) MaskRCNN with X101.

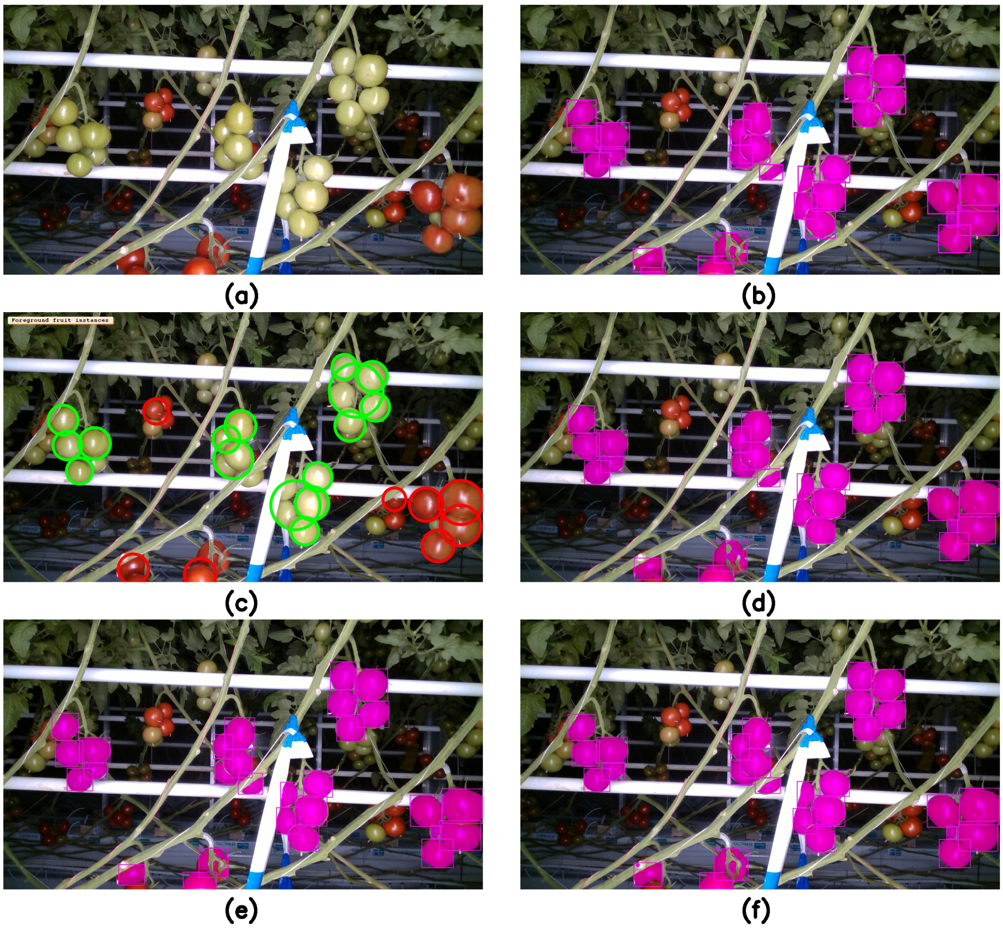

Figure S5. Camera 3 (height 2300 mm), inference with single fruit class: (a) RGB image; (b) image with overlaid ground truth; (c-f) image overlaid with fruit detection using (c) Classical segmentation using colorspaces and shape, (d) MaskRCNN with R50 architecture, (e) MaskRCNN with R101, (f) MaskRCNN with X101.

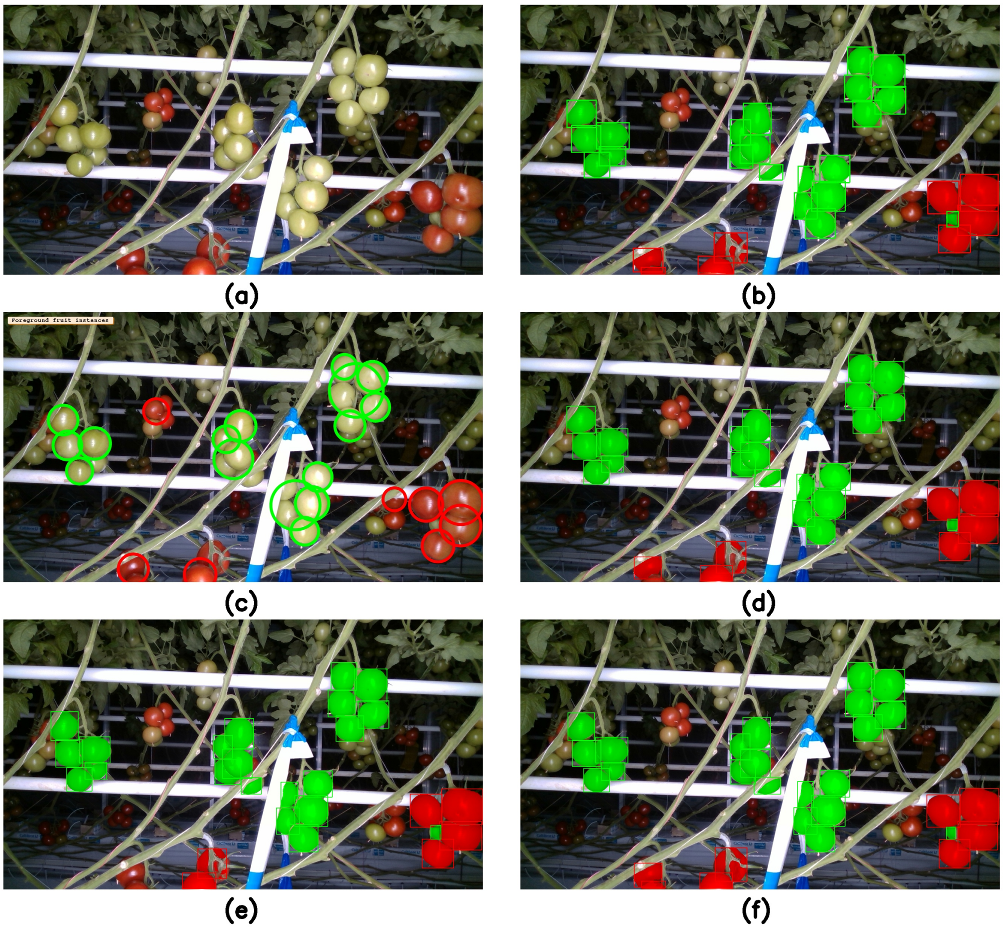

Figure S6. Camera 3 (height 2300 mm), inference with two ripeness classes: (a) RGB image; (b) image with overlaid ground truth; (c-f) image overlaid with fruit detection using (c) Classical segmentation using colorspaces and shape, (d) MaskRCNN with R50 architecture, (e) MaskRCNN with R101, (f) MaskRCNN with X101.

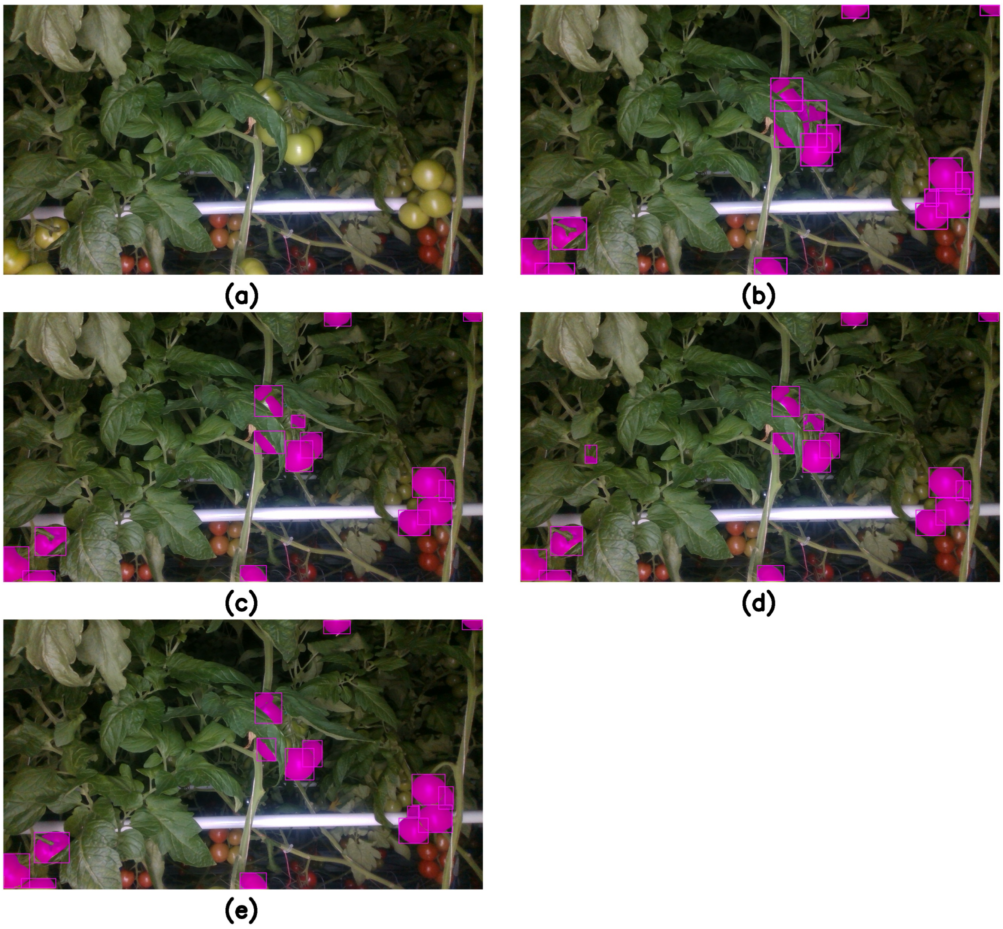

Figure S7. Camera 4 (height 3000 mm), inference with single fruit class: (a) RGB image; (b) image with overlaid ground truth; (c-f) image overlaid with fruit detection using (c) Classical segmentation using colorspaces and shape, (d) MaskRCNN with R50 architecture, (e) MaskRCNN with R101, (f) MaskRCNN with X101.

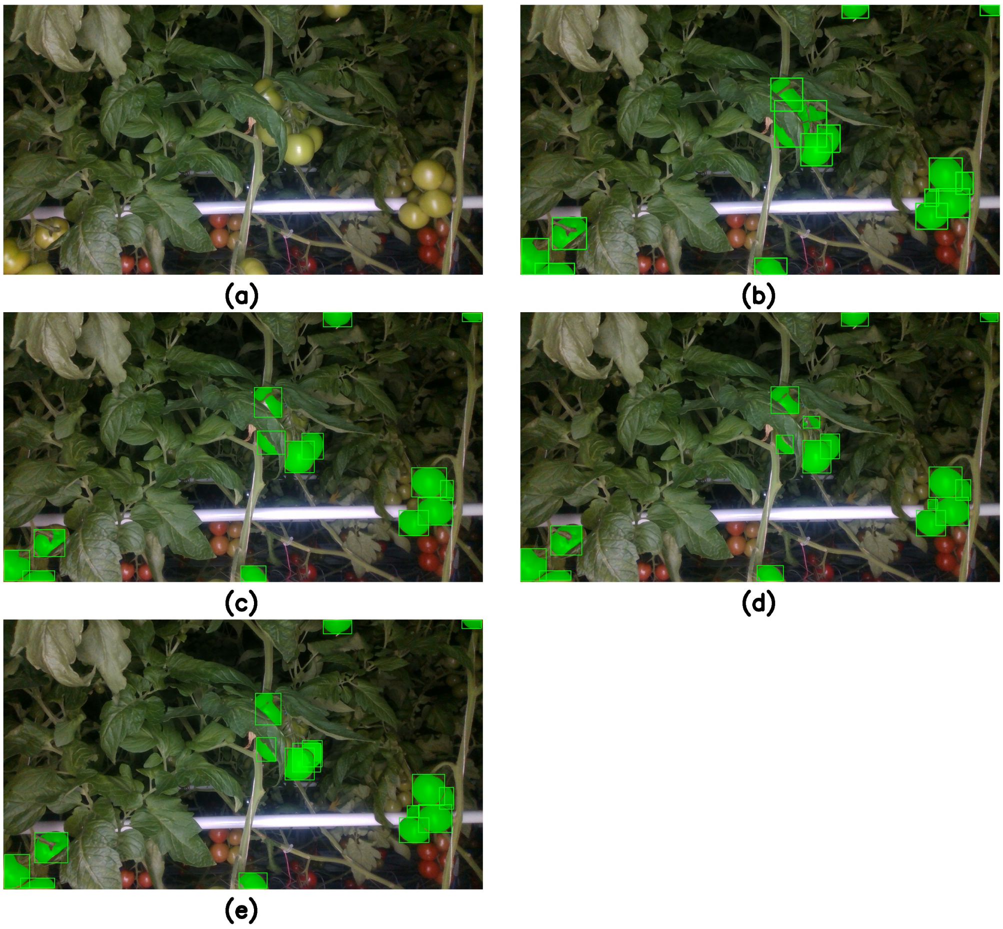

Figure S8. Camera 4 (height 3000 mm), inference with two ripeness classes: (a) RGB image; (b) image with overlaid ground truth; (c-f) image overlaid with fruit detection using (c) Classical segmentation using colorspaces and shape, (d) MaskRCNN with R50 architecture, (e) MaskRCNN with R101, (f) MaskRCNN with X101.

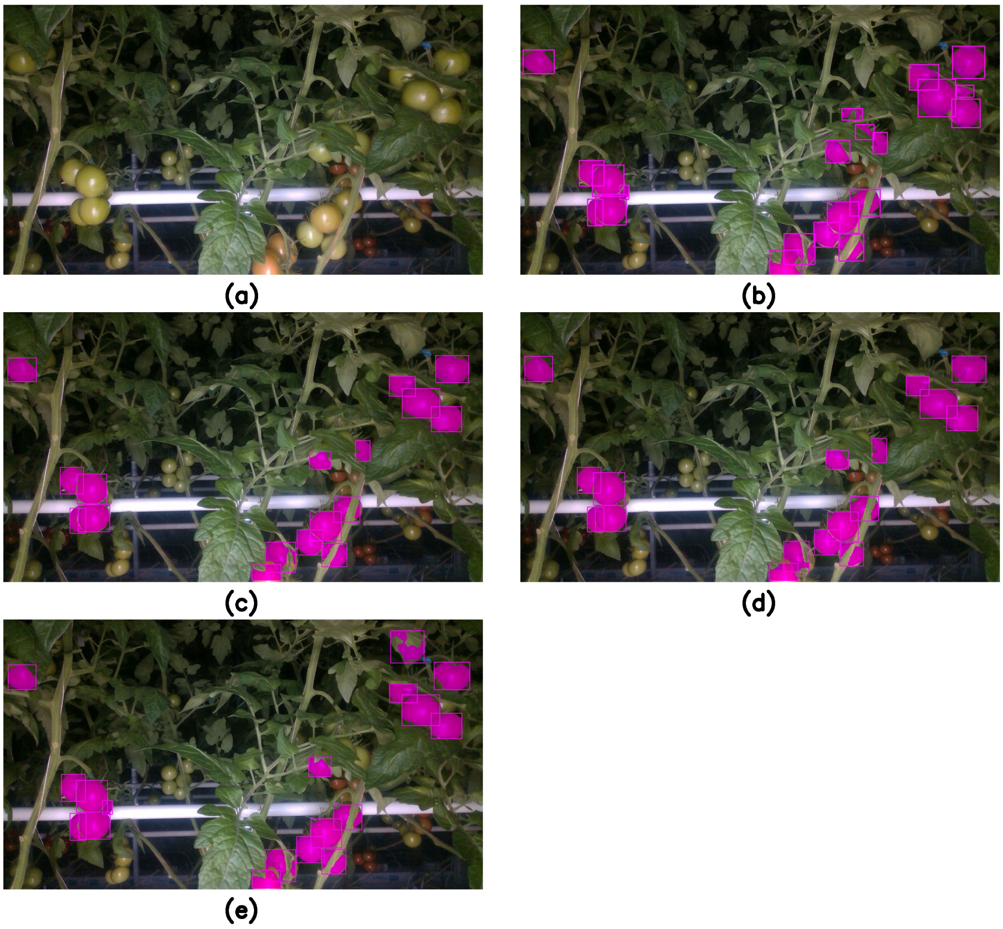

Figure S9. Camera 4 (height 3000 mm), inference with single fruit class: (a) RGB image; (b) image with overlaid ground truth; (c-f) image overlaid with fruit detection using (c) Classical segmentation using colorspaces and shape, (d) MaskRCNN with R50 architecture, (e) MaskRCNN with R101, (f) MaskRCNN with X101.

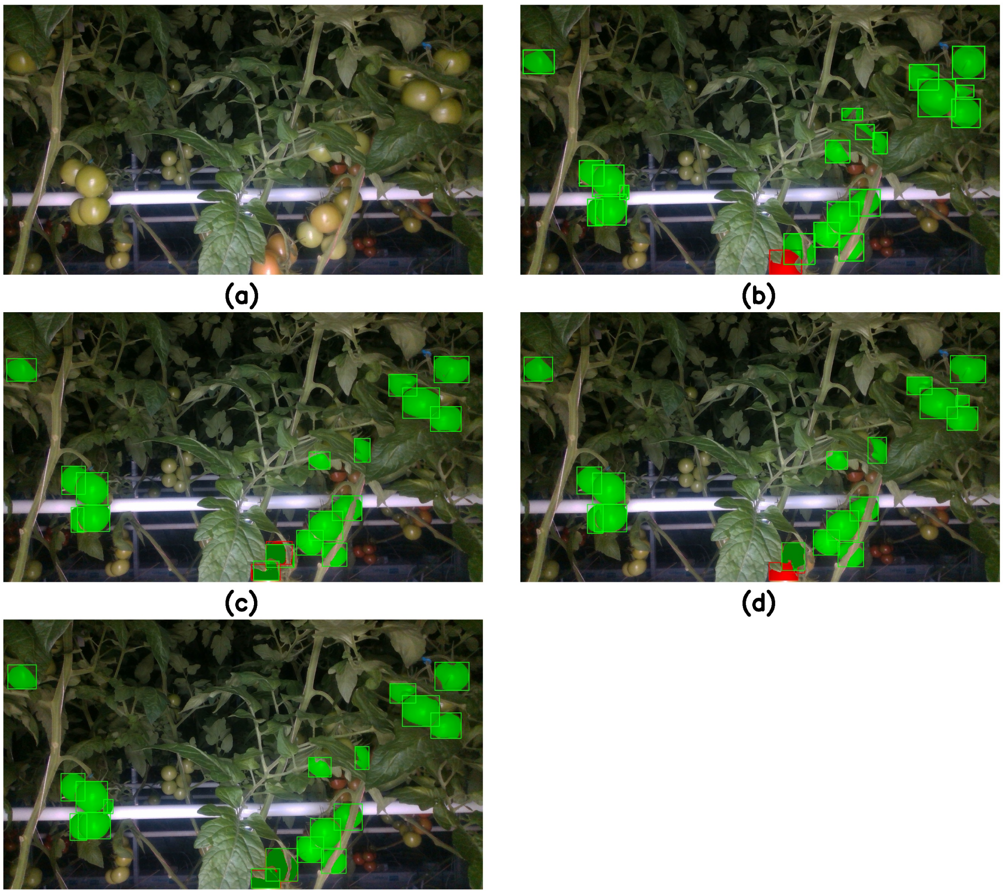

(a) (b) (c) (d) (e)

Figure S10. Camera 4 (height 3000 mm), inference with two ripeness classes: (a) RGB image; (b) image with overlaid ground truth; (c-f) image overlaid with fruit detection using (c) Classical segmentation using colorspaces and shape, (d) MaskRCNN with R50 architecture, (e) MaskRCNN with R101, (f) MaskRCNN with X101.

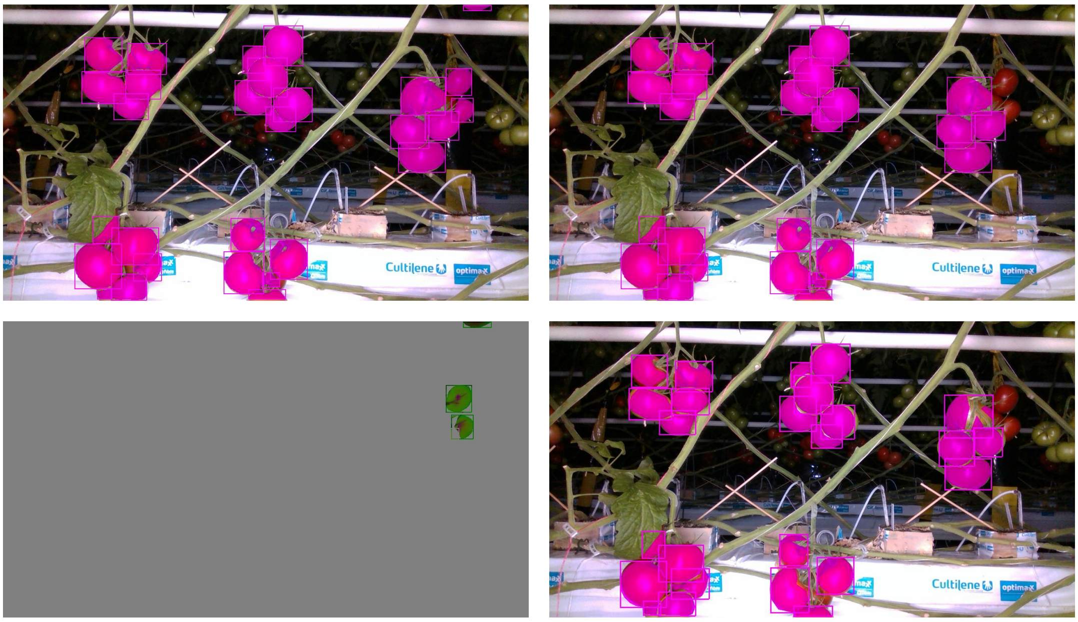

Figure S11. Example 1 - single class detection with post-processing. (top left) result of detection with ResNext101; (top right) detection after PP; (bottom left) absolute difference (tl)-(tr); (bottom right) image with overlaid ground truth. It can be seen that two false positives (background tomatoes) in the top right of the image are being removed by post-processing.

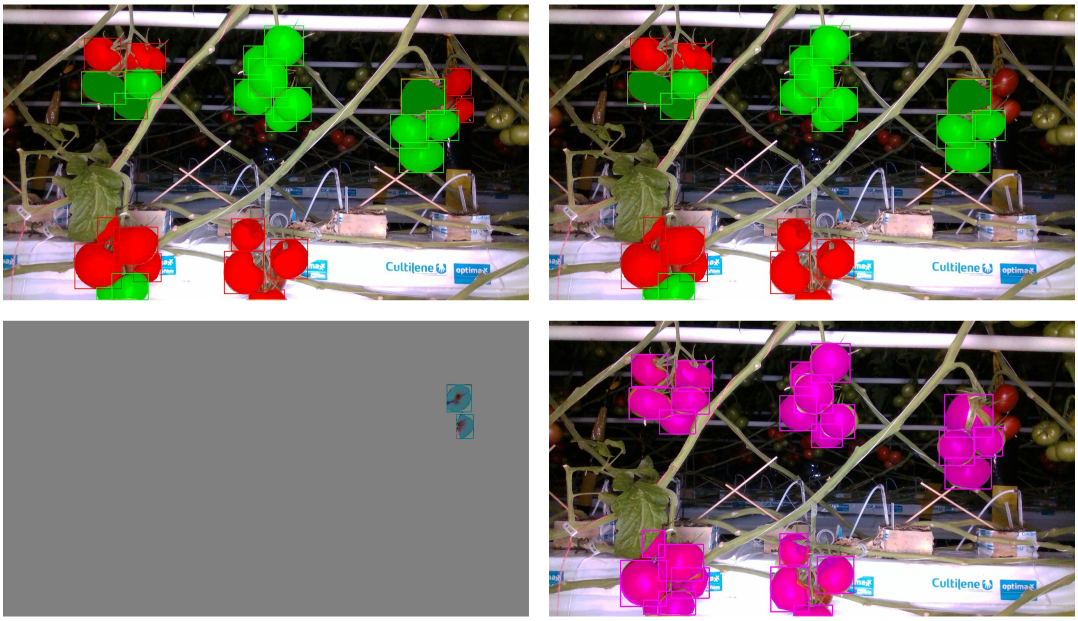

Figure S12. Example 1 - two class detection with post-processing. (top left) result of detection with ResNext101; (top right) detection after PP; (bottom left) absolute difference (tl)-(tr); (bottom right) image with overlaid ground truth. It can be seen that two false positives (background tomatoes) in the top right of the image are being removed by post-processing.

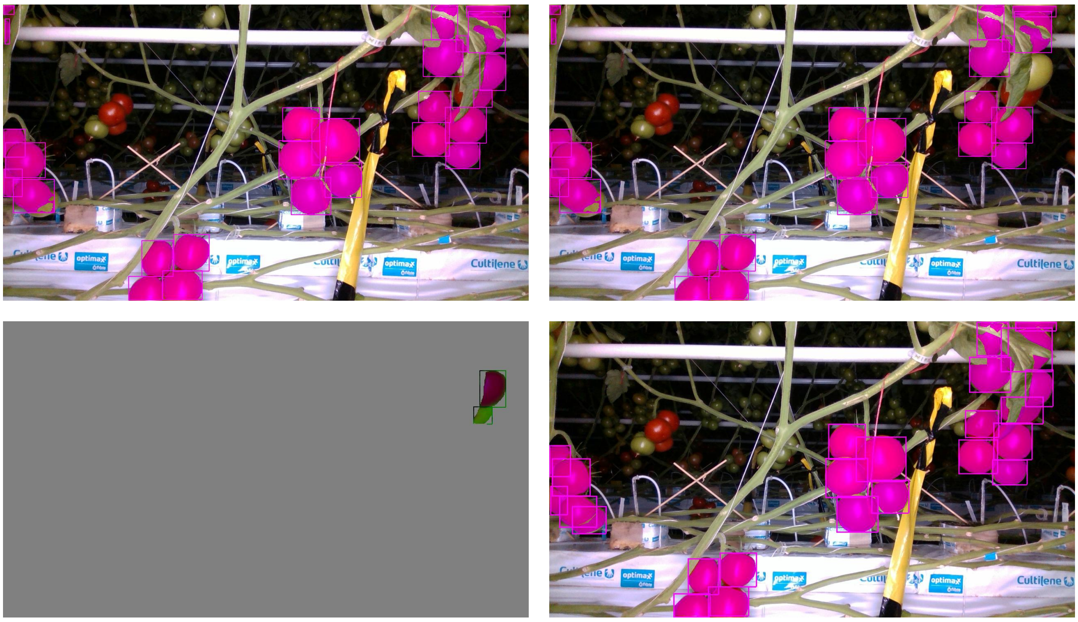

Figure S13. Example 2 - single class detection with post-processing. (top left) result of detection with ResNext101; (top right) detection after PP; (bottom left) absolute difference (tl)-(tr); (bottom right) image with overlaid ground truth. It can be seen that two true positives (foreground tomatoes) in the top right of the image are being incorrectly removed by post-processing.

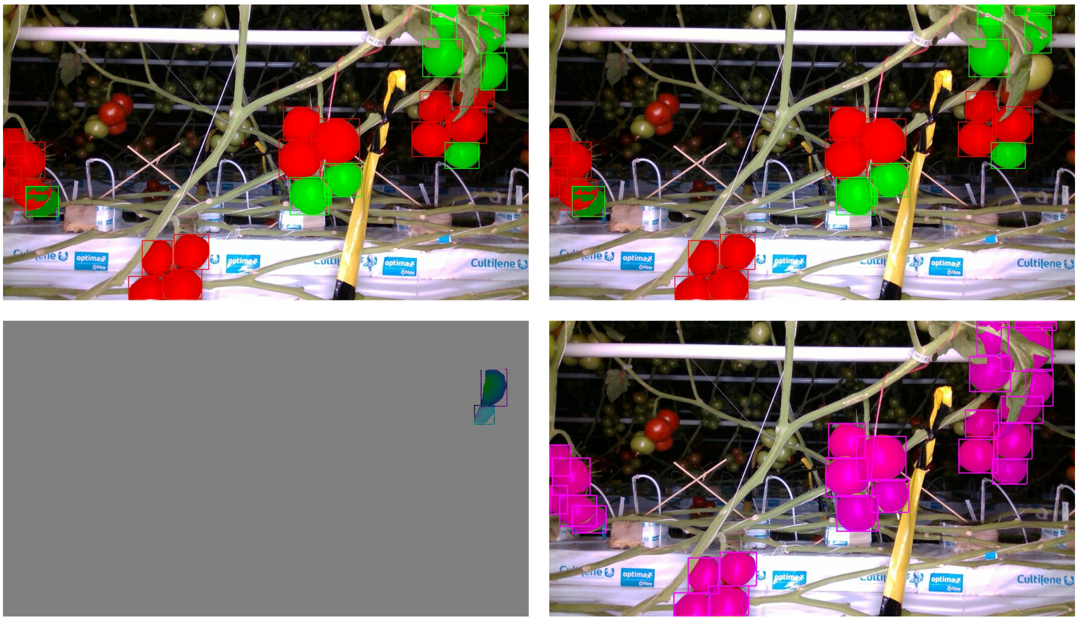

Figure S14. Example 2 - two class detection with post-processing. (top left) result of detection with ResNext101; (top right) detection after PP; (bottom left) absolute difference (tl)-(tr); (bottom right) image with overlaid ground truth. It can be seen that two true positives (foreground tomatoes) in the top right of the image are being incorrectly removed by post-processing.
